# Noradrenaline and Seizures: A Perspective on the Role of Adrenergic Receptors in Limbic Seizures

**DOI:** 10.2174/1570159X20666220327213615

**Published:** 2023-09-01

**Authors:** Francesca Biagioni, Roberta Celli, Stefano Puglisi-Allegra, Ferdinando Nicoletti, Filippo Sean Giorgi, Francesco Fornai

**Affiliations:** 1 I.R.C.C.S. Neuromed, Pozzilli, Italy;; 2 Department of Physiology and Pharmacology, University Sapienza, Rome, Italy;; 3 Human Anatomy, Department of Translational Research and New Technologies in Medicine and Surgery, University of Pisa, Pisa, Italy

**Keywords:** Area tempestas, piriform cortex, limbic seizures, noradrenaline, adrenergic receptors, β_2_-adrenergic receptors

## Abstract

**Background:**

Noradrenergic fibers originating from the locus coeruleus densely innervate limbic structures, including the piriform cortex, which is the limbic structure with the lowest seizure threshold. Noradrenaline (NA) modulates limbic seizures while stimulating autophagy through β_2_-adrenergic receptors (AR). Since autophagy is related to seizure threshold, this perspective questions whether modulating β_2_-AR focally within the anterior piriform cortex affects limbic seizures.

**Objective:**

In this perspective, we analyzed a potential role for β_2_-AR as an anticonvulsant target within the anterior piriform cortex, area tempestas (AT).

**Methods:**

We developed this perspective based on current literature on the role of NA in limbic seizures and autophagy. The perspective is also grounded on preliminary data obtained by micro-infusing within AT either a β_2_-AR agonist (salbutamol) or a β_2_-AR antagonist (butoxamine) 5 minutes before bicuculline.

**Results:**

β_2_-AR stimulation fully prevents limbic seizures induced by bicuculline micro-infusion in AT. Conversely, antagonism at β_2_-AR worsens bicuculline-induced seizure severity and prolongs seizure duration, leading to self-sustaining status epilepticus. These data indicate a specific role for β_2_-AR as an anticonvulsant in AT.

**Conclusion:**

NA counteracts limbic seizures. This relies on various receptors in different brain areas. The anterior piriform cortex plays a key role in patients affected by limbic epilepsy. The anticonvulsant effects of NA through β_2_-AR may be related to the stimulation of the autophagy pathway. Recent literature and present data draw a perspective where β_2_-AR stimulation while stimulating autophagy mitigates limbic seizures, focally within AT. The mechanism linking β_2_-AR to autophagy and seizure modulation should be extensively investigated.

## 1 INTRODUCTION

Limbic seizures are the manifestation of an epileptic activity of neurons belonging to limbic cortical structures [1]. They are the most common type of seizures in humans [2], and thus, their pathophysiology has been studied by several groups using a variety of experimental models. It is known that noradrenaline (NA) has powerful antiepileptic effects on the limbic system. This was originally evidenced by McIntyre [3-12] by using the amygdala kindled rat, where a key anticonvulsant effect was manifest for the α_2_ adrenergic receptor (AR) agonist clonidine. In line with this, damage to catecholamine-containing axons induced by 6-hydroxydopamine worsens seizures following amygdala kindling. Such an effect is replicated by administering NA antagonists, but not dopamine (DA) antagonists, which indicates the specific effect of NA compared with DA as an endogenous anticonvulsant among catecholamines [13, 14].

More recently, the effects of selective damage to NA axons arising from the locus coeruleus (LC, the main source of NA in the forebrain) induced by the neurotoxin DSP-4 have been assessed in rats undergoing focal micro-infusions of bicuculline (a selective GABA-A receptor antagonist [15]) into the anterior extent of the piriform cortex known as area tempestas (AT) [16]. This is an allo-cortical region, where neurons in the deep layer have a very low threshold to trigger seizures [17]. The AT is featured by a dense network of NA axon terminals originating from the LC [16]. Damage to these NA terminals converts serial seizures into self-sustaining status epilepticus (SE). This is concomitant with a worsening of seizure-induced damage in the limbic system [16].

In this previous study, evidence is provided that the occurrence of limbic seizures triggers a concomitant NA release within the very same limbic structures [16]. This is likely to provide an endogenous self-limiting effect, where subcortical structures involved in gating limbic seizure propagation [18] compensate for delaying seizure onset and limiting seizure propagation and duration [16]. Consistently, when damage to NA axons is produced before bicuculline is micro-infused within AT, a conversion from sporadic seizures into spontaneous, self-sustaining, long-lasting SE occurs. In this case, the increase in extracellular NA is abolished [16]. The mechanisms leading to a powerful anticonvulsant effect by endogenous NA remain to be established. A recent hypothesis claims the involvement of the autophagy pathway as a tuning mechanism for seizures threshold [19]. In line with this, a specific sub-type of adrenergic receptor (AR), β_2-_AR, was found to be key in triggering autophagy upon specific neuronal needs [20].

## 2 RESULTS AND DISCUSSION

The present perspective article addresses whether β_2_-AR subtypes are involved in limbic seizures. When investigating the role of NA as an anticonvulsant [16], we also carried out experiments on the role of β_2_-AR, which is reported for the first time in the present perspective.

In fact, β_2_-AR, which may alter seizure susceptibility through a variety of mechanisms, including ion channels modulation [21], also induces autophagy when required [20]. This mechanism was recently shown to be a pivot in modulating seizures focally induced by micro-infusions of the GABA-A antagonist bicuculline within AT [22]. Therefore, this article sheds a perspective on joining the anticonvulsant role of NA with the autophagy pathway and its potential effects in modulating seizures. At the same time, these data represent a piece of evidence that anticipates a whole ARs screening to modulate focally limbic seizures.

**Fig. (1):**
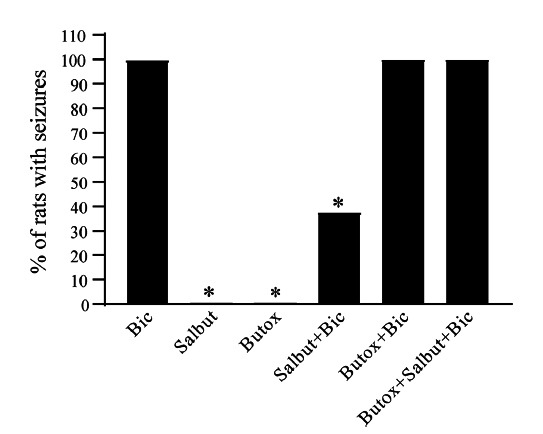
Seizure occurrence in rats micro-infused bicuculline within AT after pre-treatment with agonists or antagonists at β_2_-AR. Rats were micro-infused into the left area tempestas with bicuculline (Bic), 5 min after microinfusion of saline (“Bic” group), salbutamol (“Salbut+Bic” group) or butoxamine (“Butox + Bic” group) or butoxamine + salbutamol (“Butox+Salbut+Bic” group) into the same area, or they were infused with saline 5 minutes after microinfusion of salbutamol (“Salbut” group) or butoxamine (“Butox” group) alone into the AT. In rats that experienced seizures, the onset of limbic seizures occurred within 10 minutes after bicuculline infusion. The graph reports the % of rats undergoing seizures following various treatments in a single experiment (N=8 rats per group) (Bic=100%, N=8/8; Salbut=0%, N=0/8; Butox=0%, N=0/8; Salbut+Bic= 37.5%, N=3/8; Butox + Bic=100%, N=8/8; Butox+Salbut+Bic=100%, N=8/8). While seizures occurred in 100% of rats belonging to the groups “Bic” and “Butox+Bic”, in several rats belonging to the group “Salbut+Bic” (5/8), seizure onset was fully prevented. Rats in the groups “Salbut” and “Butox” did not show any seizures. *P < 0.05 *vs.* Bic.

In detail, these data show that in Sprague Dawley male adult rats, pre-infusion of β_2_-AR agonist salbutamol (10 nmol) prevents focally serial seizures evoked by bicuculline (118 pmol) when micro-infused 5 minutes later within AT (Fig. **1**). Conversely, such an effect is prevented by pre-infusing the selective β_2_-AR antagonist butoxamine (10 µmol) 5 minutes before bicuculline (Fig. **1**) (Supplementary File contains details on Methods).

Even in the few rats that still undergo bicuculline-induced sporadic seizures after salbutamol pre-administration, both seizure duration and seizure severity are reduced compared with rats microinfused with bicuculline alone (Table **1**). Conversely, butoxamine increases seizure number and seizure severity when administered before bicuculline or before salbutamol+bicuculline (Table **1** and Fig. **1**). These data show that AT β_2_-ARs play a key role in seizure modulation.

Remarkably, antagonism at β_2_-AR within AT does not merely worsen seizures being sometimes sufficient in converting serial seizures into self-sustaining, long-lasting SE (Table **1**). In humans, this is a medical emergency, which is associated with increased mortality in subjects affected by limbic epilepsy. These latter findings disclose a novel potential mechanism for the onset of SE and might be relevant for novel therapeutic development in SE-suffering patients. During seizures evoked from AT, a compensatory increase of NA within AT itself is observed, which, by acting at β_2_-AR, might mitigate seizure progression. When compensatory NA release cannot occur, as observed in rats lacking LC terminals [16], seizures evoked from the AT progress to SE.

Previous studies have shown a key role of AT glutamatergic AMPA receptors to convert bicuculline-triggered serial seizures into self-sustaining SE [16, 23]. Specifically, the pharmacological block of AMPA receptor desensitization by cychlothiazide [16, 23] or the over-expression of a non-desensitizing GluA1 subunit in the AT [23] fosters SE. Since the expression of non-desensitizing AMPA receptors is fostered by autophagy failure, the lack of an endogenous autophagy-inducing stimulus (NA activating β_2_-AR) may induce effects similar to those observed after blocking AMPA receptor desensitization. An additional role in this chain of events is likely to be played by mGlu5 receptors, which very recently were implicated in modulating autophagy [24].

Additional mechanisms need to be taken into account, such as β_2_-AR modulation of long-lasting phenomena related to the cascade induced by the expression of immediate early genes (genes whose protein products are transcription factors for proteins). The expression of the fos immediate early gene in the piriform cortex is specifically altered after damage to LC terminals in parallel with increased epileptogenesis [25]; this might affect the expression of glutamate receptor subunits. A reduced epileptogenic effect of AMPA receptors following β_2_-AR stimulation may depend on autophagy. In fact, recent data show that increased autophagy decreases AMPA receptor expression within limbic regions [26].

In the scenario of NA and epilepsy, one should consider that these data focus on the specific role of β-AR placed within AT, leaving uncovered the role of the very same receptors on seizure activity within different brain regions. Again, when seizures are induced through systemic administration of chemoconvulsants, even systemic effects induced by β-AR become relevant. This applies to body temperature, blood pressure, neuroendocrine and inflammatory events, as well as blood-brain barrier permeability. All these events may alter the seizure threshold within AT and the whole limbic epileptic circuitry [18].

## CONCLUSION

The anterior piriform cortex, area tempestas (AT), is a brain region that acts as a pivot in the onset, propagation, and refractoriness of limbic seizures in rodents [17]. This is relevant in humans affected by temporal lobe epilepsy [27]. Due to the remarkable preservation of neurons and neural networks through phylogenesis, pharmacological data obtained from AT in rats bear translational relevance in humans. Based on this perspective, one might infer that manipulation of β_2_-AR in patients with temporal lobe epilepsy is relevant. In fact, this might affect both seizure threshold and seizure severity, including the onset of SE. Further investigations are needed to establish the specific role of all subtypes of ARs on seizures originating from AT. As a further investigation, a link between ARs stimulation and autophagy needs to be established concerning seizure onset from AT.

## Figures and Tables

**Table 1:** Effects of β_2_-AR agonists and antagonists on seizures evoked by bicuculline microinfusion into the AT.

**Treatment**	**Time to Last Seizure (Minutes)**	**Maximum ** **Seizure Score**
Bic	47.25 ±6.9	2.5 ± 0.5
Salbut	0 *	0 *
Butox	0 *	0 *
Salbut + Bic	16.3 ±2.6 *	0.5 ± 0.71*
Butox + Bic	72 ±12.46 *	4.25 ± 1.09*
Butox + Salbut+ Bic	70.37 ± 12.5 *	4.5 ± 1.12*

Time to last seizure: time from seizure onset after microinfusion into the AT, up to end of last seizure observed during a period of at least 2 h after microinfusion.

“Maximum seizure score” is an index of seizure severity and represents the highest seizure score (see below details) reached by a seizure episode during the observation period.

Rats were microinfused into the left AT with bicuculline (Bic), 5 min after microinfusion of saline (“Bic” group), salbutamol (“Salbut+Bic” group) or butoxamine (“Butox + Bic” group) or butoxamine + salbutamol (“Butox+Salbut+Bic” group) into the same area, or they were infused with saline 5 minutes after microinfusion of salbutamol (“Salbut” group) or butoxamine (“Butox” group) alone into the AT. The severity of each seizure experienced by the rats was evaluated according to the following behavioral score modified from Racine (1972) (Fornai *et al.*, 2005; Giorgi *et al.*, 2003, 2008): 0=no seizures, 0.5=jaw clonus; 1=myoclonic jerks of contralateral forelimb; 2=mild bilateral forelimb clonus lasting 5-15 s; 3=bilateral forelimb clonus lasting>15 s; 4=rearing, with concomitant bilateral forelimb clonus; 5=rearing with loss of balance and concomitant forelimb +/− hind limb clonus. The onset of status epilepticus (SE) was also recorded, which was assigned a score of 6. SE is defined here as convulsive seizure activity lasting, without any interruption, for at least 5 minutes. SE occurred in 2/8 of rats in both the Butox+Bic group and Butox+Salbut+Bic group, while it never occurred in any one of the other groups. *P < 0.05 *vs*. Bic.
