# Supplementary material for: Noradrenaline and Seizures: A Perspective on the Role of Adrenergic Receptors in Limbic Seizures
Source: Curr Neuropharmacol. 2023 Sep 1;21(11):2233–6. doi: 10.2174/1570159X20666220327213615 (PMC10556380; doi:10.2174/1570159X20666220327213615)
Supplement: Supplementary file 1 — Supplementary material is available on the publisher’s website along with the published article. [file CN-21-2233_SD1.pdf]

Supplementary Material

Noradrenaline and Seizures: A Perspective on the Role of Adrenergic Receptors in Limbic Seizures

Francesca Biagioni[1], Roberta Celli[1], Stefano Puglisi-Allegra[1], Ferdinando Nicoletti[1,2], Filippo Sean Giorgi[3] and Francesco Fornai[1,3,*]

[1]I.R.C.C.S. Neuromed, Pozzilli, Italy; [2]Department of Physiology and Pharmacology, University Sapienza, Rome, Italy; [3]Human Anatomy, Department of Translational Research and New Technologies in Medicine and Surgery, University of Pisa, Pisa, Italy

1875-6190/23 $65.00+.00 **© 2023 Bentham Science Publishers**

METHODS

Surgery, Seizure Induction and Seizure Assessment

Adult male Sprague Dawley rats weighing 280-320 g were submitted to stereotactic surgery under deep (Cloral Hydrate 400 mg/kg) anesthesia, for placement of a guide 22-gauge cannula. The experiments reported in this perspective have been performed in concomitance with the approved protocol for the experiments outlined by Giorgi *et al.* (2003). Twenty-four hours after surgery, an injection cannula (27-gauge) allowing infusion of chemoconvulsants was transiently inserted within the guide cannula to reach the anterior extent of left deep piriform cortex (Area Tempestas, AT), whose coordinates were AP=+4 mm from the bregma, ML=+3.2 mm from the midline and DV=−6.5 mm below the dura (according to the atlas of Pellegrino *et al.* 1979). The injection cannula was connected by polyethylene tube to a Hamilton syringe operated automatically by a Sage infusion pump with an infusion rate of 60 nL/minute. Further details on surgery are reported by Giorgi *et al.* (2003).

Bicuculline (118 pmol in 120 nL, Sigma Aldrich, Milan, Italy) was microinfused for 2 minutes into the AT. Five minutes before Bicuculline microinfusion rats were microinfused with saline (120 nL) ("Bic"group), or salbutamol (10nmol in 200 nL, Sigma)("Salbut+Bic" group) or butoxamine (10 µmol in 120 nL, Sigma) ("Butox+Bic" group) or both ("Butox+Salbut+Bic" group) (doses inferred and in line with exisiting literature and receptor affinity; Capuano *et al.*, 1992; Waldmeier, 1981; Lazzeri *et al.*, 2021). The amount of each compound to be micro-infused is expressed in number of moles (mol), which corresponds to the number of authentic molecules of each compound, according to the mole X the Avogadro number (moles X 6.022 X 10^{23}). This unit of measure is routinely applied in manuscripts expressing the amount of drugs micro-infused in the piriform cortex, area tempestas, starting from the pioneer paper by Piredda and Gale (1985).

In two groups of rats, animals were microinfused into the AT by either butoxamine or salbutamol, and five minutes later, they were infused with saline (120 nL) rather than bicuculline ("Butox" and "Salbut" groups, respectively). Each microinfusion within the AT was carried out for two minutes. After the end of each infusion, the infusion cannula was left in place for one more minute in order to allow complete delivery of the substance into the AT. Rats were observed individually for up to 1.5 hours (or after the last seizure episode occurrence) after infusion by an operator blinded to treatment; maximum seizure severity was scored using a 0.5-6 score according to Fornai *et al.*, 2005 (Fig. **1**). Only rats in which the correct cannula placement into the AT was verified after sacrifice were used for data analysis (N=8 rats for each group).

The following seizure data were compared between groups: a) percentage of rats experiencing at least one seizure episode after bicuculline; b) time from the infusion up to the end of the last seizure episode recorded by behavioral observation; c) maximum seizure score of the seizures experienced by each animal.

Statistical Analysis

Comparisons of the percentage of animals experiencing seizures within each group were performed by Chi Square analysis. Comparisons among the different groups concerning seizure severity (maximum seizure score) and seizure duration (min) were performed by Kruskal-Wallis analysis with Mann-Whitney post-hoc analysis.

Null hypothesis was rejected for $P < 0.05$.

RELATED REFERENCES

- Capuano CA, Leibowitz SF, Barr GA. The pharmaco-ontogeny of the perifornical lateral hypothalamic beta 2-adrenergic and dopaminergic receptor systems mediating epinephrine- and dopamine-induced suppression of feeding in the rat.

Brain Res Dev Brain Res. 1992 Nov 20;70(1):1-7.

- Giorgi FS, Ferrucci M, Lazzeri G, Pizzanelli C, Lenzi P, Alessandrì MG, Murri L, Fornai F. A damage to locus coeruleus neurons converts sporadic seizures into self-sustaining limbic status epilepticus. Eur J Neurosci. 2003 Jun;17(12):2593-601.
- Lazzeri G, Busceti CL, Biagioni F, Fabrizi C, Morucci G, Giorgi FS, Ferrucci M, Lenzi P, Puglisi-Allegra S, Fornai F. Norepinephrine Protects against Methamphetamine Toxicity through β2-Adrenergic Receptors Promoting LC3 Compartmentalization. Int J Mol Sci. 2021 Jul 5;22(13):7232. doi: 10.3390/ijms22137232.
- Piredda S, Gale K. A crucial epileptogenic site in the deep prepiriform cortex. Nature. 1985 Oct 17-23;317(6038):623-5
- Waldmeier PC. Stimulation of central serotonin turnover by beta-adrenoceptor agonists. Naunyn Schmiedebergs Arch Pharmacol. 1981 Sep;317(2):115-9. doi: 10.1007/BF00500065.
